# Active Inference Through Energy Minimization in Multimodal Affective Human–Robot Interaction

**DOI:** 10.3389/frobt.2021.684401

**Published:** 2021-11-26

**Authors:** Takato Horii, Yukie Nagai

**Affiliations:** ^1^ Graduate School of Engineering Science, Osaka University, Osaka, Japan; ^2^ International Research Center for Neurointelligence (WPI-IRCN), The University of Tokyo, Tokyo, Japan; ^3^ Institute for AI and Beyond, The University of Tokyo, Tokyo, Japan

**Keywords:** active inference., energy based models, emotion, human-robot interaction, multimodal perception

## Abstract

During communication, humans express their emotional states using various modalities (e.g., facial expressions and gestures), and they estimate the emotional states of others by paying attention to multimodal signals. To ensure that a communication robot with limited resources can pay attention to such multimodal signals, the main challenge involves selecting the most effective modalities among those expressed. In this study, we propose an active perception method that involves selecting the most informative modalities using a criterion based on energy minimization. This energy-based model can learn the probability of the network state using energy values, whereby a lower energy value represents a higher probability of the state. A multimodal deep belief network, which is an energy-based model, was employed to represent the relationships between the emotional states and multimodal sensory signals. Compared to other active perception methods, the proposed approach demonstrated improved accuracy using limited information in several contexts associated with affective human–robot interaction. We present the differences and advantages of our method compared to other methods through mathematical formulations using, for example, information gain as a criterion. Further, we evaluate performance of our method, as pertains to active inference, which is based on the free energy principle. Consequently, we establish that our method demonstrated superior performance in tasks associated with mutually correlated multimodal information.

## 1 Introduction

Humans use signals of various modalities to communicate their internal states to one another. For instance, when interacting, humans use facial expressions, gestures, and vocalizations to express their emotions and to perceive the emotions of others. Complex relationships exist between such multimodal signals. Sometimes, such multimodal signals demonstrate correlative relationships, and at other times, they exhibit complementary characteristics. Specifically, multimodal expressions of emotion have strong interrelations because the emotional states of humans are linked to their bodies and are widely expressed in their multimodal signals. Therefore, it is necessary to select informative signals to estimate the emotions of others accordingly.

Various researchers have proposed the development of communication robots that can estimate human emotions. Breazeal and Aryananda (2002) used acoustic features to determine the emotional states of interaction partners. Barros et al. (2015); Elfaramawy et al. (2017) used facial expressions and gestures as visual signals for emotion recognition. In contrast, Breazeal (2003); Watanabe et al. (2007); Lim and Okuno (2014); Barros and Wermter (2016) focused on multimodal expressions to recognize the emotional states of interaction partners. For instance, Lim and Okuno (2014) developed a communication robot that was able to acquire multimodal representations of human emotions using human voice and gait. Barros and Wermter (2016) developed a multimodal deep neural network that uses audio-visual signals to recognize the emotional state of humans. However, a robot cannot always deal with all the available modality information simultaneously. Thus, it must actively access the dynamically changing emotional expressions of the interaction partner instead of accessing all the information of the interaction partner over time. We believe that it is necessary to estimate emotions using as little information as possible owing to the inherent resource limitations of robotic systems and inevitably continuous changes in an interaction partner’s emotional state during the interaction process. Moreover, not every modality signal meaningfully indicates the actual state of a partner because some signals may contain noise or ambiguity. The robot should select the most informative modalities among those available to estimate the target states.

In the field of robotics, the issue of attention control for obtaining information to update estimations is formulated as active perception. In many studies, the attention point of a robotic camera has been controlled or actions have been selected to perceive sensory signals (Sakaguchi, 1993; Roy et al., 2004; Chen et al., 2011). For instance, Taniguchi et al. (2018) proposed an active perception strategy designed to determine the order of perception for multimodal signals (e.g., vision, audio, and tactile signals) in an object recognition task. The proposed method involved selecting a modality that maximized information gain (see Section 2 for details). However, we hypothesize that there is a large gap between object recognition and emotion estimation. Taniguchi et al. (2018) assumed that the multimodal signals from an object were independent of each other. In contrast, multimodal signals relating to human emotions may have complex interrelationships with one another. Few works have considered the relative effectiveness of active perception methods for modality selection during emotion estimation.

In neuroscience, action control for obtaining perception is investigated as active inference. Active inference (Friston et al., 2017) is an action selection method based on the free energy principle, which is a fundamental principle related to the human brain. The key concept underlying active inference is that the human brain performs actions to reduce the prediction error between the state of the environment (i.e., outside the brain) and state prediction. From this perspective, the attention shift in multimodal signals can be regarded as the action execution to switch modalities to reduce the estimation uncertainty. Recently, some researchers have studied the relationship between active inference and other algorithms, such as reinforcement learning, active learning, and control as inference (Hafner et al., 2020; Imohiosen et al., 2020). Based on the aforementioned studies, we consider that the neural mechanism of active inference also accounts for active perception.

**FIGURE 1:**
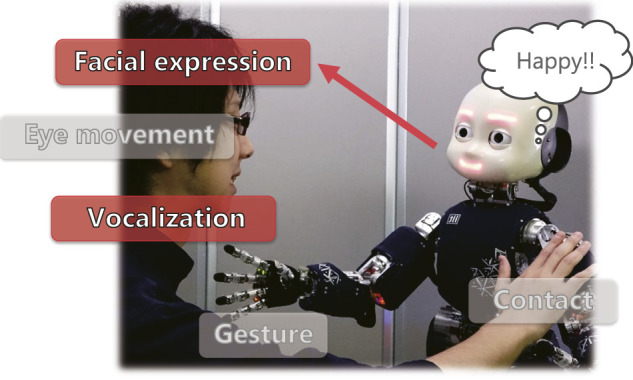
Action selection during multimodal affective interaction between a human and robot.

The objective of our research is to apply the concept of active inference to estimate the emotions of humans in multimodal human–robot interactions, as shown in Figure 1. We propose an active perception method based on expected energy minimization in an energy-based model. This model represents the joint probabilities of observation and latent variables according to an energy value function. A lower energy value corresponds to a higher probability (i.e., lower uncertainty) of the data in the proposed model. Therefore, the proposed approach enables active perception by minimizing the expected energy, which is calculated from the predicted unobserved modalities, among all selectable modalities. Moreover, it employs a multimodal deep belief network (MDBN), which is an energy-based model, and acquires a shared representation among multimodal signals (Ngiam et al., 2011; Horii et al., 2016, 2018). For instance, Ngiam et al. (2011) fused audio-visual signals using a bimodal DBN to estimate spoken digits and letters from human speech. We use an MDBN to learn the relationship between the multimodal expressions of humans and their emotional states by abstracting and integrating multimodal signals. As a first step in this study, we used the IEMOCAP dataset, which is a multimodal human–human interaction dataset, to train the MDBN. We then evaluated the proposed active perception method on its ability to perform human emotion estimation. The experimental results show that the proposed method achieved higher accuracy using less information than other active perception methods for emotion estimation.

Finally, we discuss the relationship between the proposed active perception method based on energy minimization and active perception based on information gain maximization from the perspective of expected free energy minimization, which is a key component of the active inference theory.

The remainder of this paper is organized as follows. Section 2 presents related work on robotics and neuroscience. Section 3 introduces energy-based models and their characteristics. Section 4 outlines and describes the mathematical formulation of our method based on energy minimization. Section 5 provides the details of the dataset and experimental settings. Subsequently, Section 6 presents the experimental results and discusses the difference between the proposed active perception method and others based on the results and mathematical formulations. Finally, Section 7 provides our final conclusions and suggests some issues to be addressed in future research.

## 2 Related Work

### 2.1 Active Perception in Robotics

Many researchers have investigated active perception, which is an important skill for robots that interact with objects, humans, and environments. The most popular application of active perception in robotics is active vision, in which a robot controls its attention to obtain information (Roy et al., 2004; Chen et al., 2011; Valipour et al., 2017; Zaky et al., 2020). For instance, Roy et al. (2005) proposed a 3D object recognition system using a single camera. The system iteratively determined the view of an object, which could not be captured by the camera initially, based on the probability of a hypothesis regarding the object. When the probability was lower than the predetermined threshold, the system determined an optimal movement to maximize the increase in the probability by obtaining another observation. Deinzer et al. (2009) proposed an active vision system that selectively moved a camera around an object. The viewpoint planning method involved reinforcement learning and selected the next viewpoint based on the information gains of candidate viewpoints.

Several studies have been conducted on active object recognition based on not only visual perception, but also tactile perception (Sakaguchi, 1993; Tanaka et al., 2014; Scimeca et al., 2020). Sakaguchi (1993) proposed an active haptic sensing system that used various tactile sensors (e.g., pressure sensors, thermal sensors, and vibration sensors) to estimate object categories. Their proposed method involved selecting the next sensor from the set of tactile sensors to maximize the mutual information between the object category and the *i*-th sensory signal. The mutual information indicated the degree with which the uncertainty of the object category estimates would be reduced when the system perceived the object using the *i*-th sensor. Their proposed method exhibited better performance than a random selection strategy, improved the recognition accuracy, and reduced the number of observations.

Taniguchi et al. (2018) proposed an active perception method using a multimodal hierarchical Dirichlet process (MHDP) for object recognition. The MHDP represented the relationships between multimodal sensory signals and object categories utilizing a probabilistic model. Their active perception method, which was formulated terms of in information theory, involved selecting the next perception modality that maximized the information gain between the current belief of an object and the expected sensory signal of each unobserved modality. They showed that their proposed method estimated object categories using fewer modalities (i.e., faster) than other methods.

In the studies mentioned above, in which object categories were estimated using single or multimodal sensors, it was assumed that the sensory signals were independent between modalities and/or observations. This assumption helped simplify the representation of the relationships between the object categories and sensory signals as well as the calculations of the mutual information and information gain. In contrast, we supposed that this assumption does not hold in emotion recognition because the multimodal signals that are expressed to convey emotions have strong interrelations. For instance, when a person speaks in a loud voice, it is expected that their mouth would open widely, and that more gestures are made than when a person speaks softly. These characteristics can help a robot decide which modality signal to perceive to update the estimation belief. Therefore, it is important to avoid assuming independence between sensory signals for emotion recognition.

### 2.2 Active Inference and Free Energy Principle

Attention selection, such as active vision and active perception, is an important cognitive function for humans as well as robots. The attention selection mechanism of humans that involves active perception has been discussed recently from the perspective of active inference (Friston et al., 2017). Active inference is one of the inference mechanisms in the free energy principle. Friston (2010) proposed that the human brain minimizes the variational free energy required to model and understand the world and that the process is realized in two ways: perceptual and active inference. Perceptual inference is the ability to infer the latent state of the stimuli evoked in the environment using the stimulus predictions and the errors between the actual and predicted stimuli. This ability is known as prediction error minimization in perception (Friston and Kiebel, 2009). In contrast, active inference refers to inference of the latent state by executing or optimizing actions to change perceptions (Friston et al., 2017). In other words, the human brain updates its estimations and reduces the uncertainty of its predictions by performing its own actions. Essentially, active inference in a set of discretized actions is related to the active perception studied by Sakaguchi (1993); Taniguchi et al. (2018).

Recently, the free energy principle and the concept of active perception have been employed in numerous investigations. One active research area considers emotions. Human emotions have been well discussed in terms of the free energy principle and active inference with embodied signals (i.e., interoception) (Seth, 2013; Seth and Friston, 2016; Barrett, 2017). Seth (2013) and Seth and Friston (2016) described the determination of the emotional states of humans as the prediction of self-body signals through, e.g., interoception. Interoception is the perception of the sensory signals of organs and hormones; thus, a sensation represents the internal state of the body. Barrett (2017) proposed an embodied predictive interoception coding model to represent human emotions based on predictive coding (i.e., the free energy principle). In this model, the emotional state is represented based on the prediction of interoception with proprioception and exteroception, and the human reaction to emotional change (e.g., paying attention to specific sensory signals) is considered an active inference for minimizing the prediction error of interoception.

Several studies on robotics and computational modeling have suggested cognitive function frameworks based on the free energy principle and active inference (Smith et al., 2019; Demekas et al., 2020; Ohata and Tani, 2020; Oliver et al., 2021). For instance, Smith et al. (2019) proposed an active inference model that learned emotional concepts and inferred emotions from simulated multimodal sensations (i.e., exteroceptive, proprioceptive, and interoceptive sensations). The proposed model performed attention selection to a valence (i.e., positive or negative) state to gain precise information from the environment. Oliver et al. (2021) proposed an active inference model for a robot to recognize its body, whereby the robot sampled a sensory signal that matched its prediction. Their model outperformed the classical inverse kinematics model in a reaching task involving real-world interaction. However, these active inference models in robotics have yet to be applied to human–robot interaction in an affective context, e.g., emotion estimation.

## 3 Energy-Based Models for Representing Emotions From Multimodal Expressions

**FIGURE 2:**
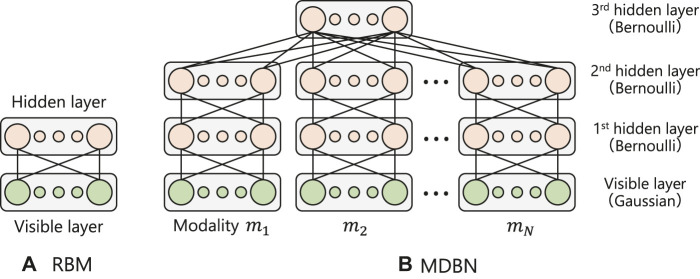
Structures of the RBM, DBN and proposed MDBN.

This section introduces a multimodal neural network called MDBN (Ngiam et al., 2011; Horii et al., 2016, 2018) designed to represent relationships between human emotions and their multimodal expressions. The MDBN is a hierarchical and multimodal extension model of a restricted Boltzmann machine (RBM) (Hinton and Salakhutdinov, 2006; Hinton, 2010), which is an energy-based model that abstracts input signals in an unsupervised manner. To compress and integrate multimodal signals, the MDBN comprises two parts, including a DBN (Hinton and Salakhutdinov, 2006) for handling each modality signal and an RBM for gathering the output of each DBN as the top layer of the model. Figure 2A,B illustrates the structures of the RBM and MDBN, respectively. In this section, we will first describe the RBM as a component of the MDBN. Next, we will explain the MDBN and its energy function, which is used in our active inference method.

### 3.1 Restricted Boltzmann Machine

An RBM (Hinton and Salakhutdinov, 2006; Hinton, 2010) is a two-layered stochastic neural network (see Figure 2A) in which each layer is composed of different types of neurons. *v*_*i*_ represents the activation of the *i*-th visible layer unit that receives external signals as inputs, and *h*_*j*_ represents the activation of the *j*-th hidden layer unit that does not receive external signals. The connecting weights between the layers are symmetric (i.e., *w*_*ij*_ = *w*_*ji*_), whereas there are no connections between units in the same layer. The RBM learns the probability of input signals in the visible layer and their abstracted representations in the hidden layer in an unsupervised manner. The joint probability of activations ***v*** and ***h*** in the RBM is represented using a Boltzmann distribution, as follows.

$$p\left(v,h;\theta \right)=\frac{1}{Z\left(\theta \right)}\exp\left(-E\left(v,h;\theta \right)\right),$$ (1)

where Z (***θ***) is a partition function and *E* (***v***, ***h***; ***θ***) is the energy function, which assigns the energy value for the corresponding activations based on the network parameter ***θ*** (i.e., connecting weights and biases of neurons). Eq. 1 represents the joint probability of the network state, thereby indicating that a lower energy state has a higher probability than a higher energy state.

The training algorithm of RBMs, contrastive divergence algorithm (Hinton, 2010), can be described as a minimization of a reconstruction error between the actual input signals and reconstructed signals from the hidden activations by modulating the parameter ***θ***. This process maximizes the joint probabilities of training data through the minimization of energy values of the data in the RBM. Finally, the energy-based model can represent the likelihood of any combination of ***v*** and ***h*** using the energy function. The reader can refer to Hinton (2010); Cho et al. (2011) for details of the update rules for the model parameters.

The activation of the stochastic unit in each layer is modeled in specific distribution (e.g., Bernoulli and Gaussian). For instance, a Bernoulli–Bernoulli RBM handles only binary signals for both the visible and hidden units (i.e., *v*_*i*_ ∈ {0, 1} and *h*_*j*_ ∈ {0, 1}). The probabilistic functions of activation for these units are given by

$$p\left(v_{i}=1|h;\theta \right)=sig\left(\underset{j}{\sum }h_{j}w_{ij}+a_{i}\right),$$ (2)

$$p\left(h_{j}=1|v;\theta \right)=sig\left(\underset{i}{\sum }v_{i}w_{ij}+b_{j}\right),$$ (3)

where ***θ*** = {***a***, ***b***, ***w***} are the model parameters, *a*_*i*_ and *b*_*j*_ are the bias parameters for the *i*-th visible and the *j*-th hidden units, respectively, and sig(*x*) is a sigmoid function 1/(1 + exp (−*x*)). The energy function of the Bernoulli–Bernoulli RBM is expressed as follows.

$$E\left(v,h;\theta \right)=-\underset{i,j}{\sum }v_{i}h_{j}w_{ij}-\underset{i}{\sum }a_{i}v_{i}-\underset{j}{\sum }b_{j}h_{j}.$$ (4)

In addition, to handle the continuous values of sensory signals in the visible layer, the binary units can be replaced with Gaussian units. The activation probabilities for the visible and hidden units of a Gaussian–Bernoulli RBM are expressed as follows.

$$p\left(v_{i}=v|h\right)=N\left(v|\underset{j}{\sum }h_{j}w_{ij}+a_{i},\sigma_{i}^{2}\right),$$ (5)

$$p\left(h_{j}=1|v\right)=sig\left(\underset{i}{\sum }\frac{1}{\sigma_{i}^{2}}v_{i}w_{ij}+b_{j}\right),$$ (6)

where N (⋅|*μ*, *σ* ^2^) denotes the probability of a Gaussian distribution with mean *μ* and variance *σ* ^2^ and *σ*_*i*_ is the standard deviation associated with the *i*-th Gaussian visible unit. The probability function of the hidden units is different from that in Eq. 3, because of the effect of the variance of the visible units. Both Bernoulli–Bernoulli and Gaussian–Bernoulli RBMs not only abstract the input signals to latent signals using Eqs 3, 6, but also reconstruct the input signals from the latent signals using Eqs 2, 5, respectively.

### 3.2 Multimodal Deep Belief Network

To acquire the relationships between human emotions and their multimodal expressions using the energy-based model, we constructed a hierarchical and multimodal extension model based on RBM methods. First, we stacked RBMs to abstract sensory signals hierarchically in each modality. A multi-stacked RBM with directed connections is called a DBN (Hinton and Salakhutdinov, 2006) where the hidden layer of a lower RBM is connected to the visible layer of an upper RBM (see Figure 2B). We employed two different types of layers to construct the DBN, including a visible layer with Gaussian distribution to take into consideration continuous sensory values, and a hidden layer with Bernoulli distribution to encode them into discrete representations. The DBN is trained for each layer independently using the contrastive divergence algorithm in an unsupervised manner.

Next, we added another hidden layer (3rd hidden layer) to associate abstracted modality signals by each DBN. The top layer of each modality DBN (2nd hidden layer) was connected to the third hidden layer, as shown in Figure 2B. Here, we assumed that humans use *N* kinds of modalities (i.e., **M** = {*m*_1_, …, *m*_*n*_, …, *m*_*N*_}, |**M**| = *N*), such as facial expressions, vocalization, and gestures to express their emotions (Figure 1). Let ${h_{n}}^{2}\in {\left\{0,1\right\}}^{J_{n}}$ denote the activation of the *n*-th modality (*m*_*n*_) DBN’s second hidden layer. We then calculated the activation probability of the *s*-th unit *z*_*s*_ ∈ {0, 1} of the third hidden layer by replacing ***v*** in Eq. 3 with $h^{2}=\left\{{h_{m_{1}}}^{2}⊕{h_{m_{2}}}^{2}⊕\cdots ⊕{h_{m_{N}}}^{2}\right\}$ (here, ⊕ denotes a concatenate operator).

$$p\left(z_{s}=1|h^{2}\right)=sig\left(\overset{J_{1}}{\underset{j}{\sum }}h_{m_{1},j}^{2}w_{js}+\cdots +\overset{J_{N}}{\underset{j}{\sum }}h_{m_{N},j}^{2}w_{js}+c_{s}\right),$$ (7)

where *w*_*js*_ is the connection weight between the *j*-th unit of each top layer of DBNs and the *s*-th unit of the third layer, and *c*_*s*_ is a bias parameter. Finally, the energy function of the second and third hidden layers that we used as the criterion for the proposed active perception method is expressed as follows.

$$\begin{array}{cc} E\left(h^{2},z;\theta \right)= & -\overset{J_{1}}{\underset{j}{\sum }}b_{j}h_{m_{1},j}^{2}-\overset{J_{1}}{\underset{j}{\sum }}\underset{s}{\sum }h_{m_{1},j}^{2}z_{s}w_{js}-\cdots \\ & -\overset{J_{N}}{\underset{j}{\sum }}b_{j}h_{m_{N},j}^{2}-\overset{J_{N}}{\underset{j}{\sum }}\underset{s}{\sum }h_{m_{N},j}^{2}z_{s}w_{js}\\ & -\underset{s}{\sum }c_{s}z_{s}. \end{array}$$ (8)

## 4 Active Perception Based on Energy Minimization in an MDBN

This section introduces the proposed active perception method based on energy minimization in the MDBN. Section 4.1 provides the details of the proposed algorithm, and Section 4.2 formulates the proposed method from the perspective of the maximization of the energy difference between the current and predicted energy values.

### 4.1 Proposed Active Perception in the Multimodal Model

The essential concept underlying our method is that a robot selects a single modality that minimizes the expected energy using the predicted unobserved sensory signals. As described in Section 3, the network energy corresponds to the likelihood of the model state. In other words, the modality that results in the lowest energy is expected to have the highest likelihood in the current estimation.

**FIGURE 3:**
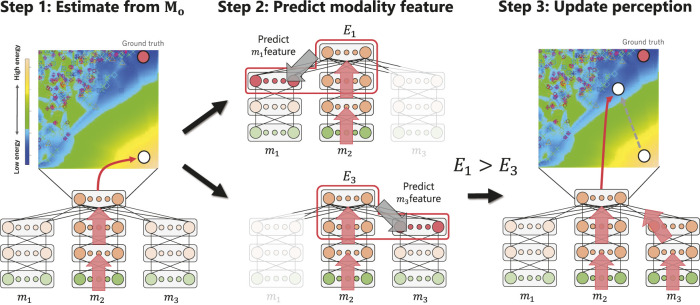
Outline of the active perception method based on energy minimization of the prediction.

Figure 3 illustrates the active perception process, assuming that the human partners use three modalities (i.e., **M** = {*m*_1_, *m*_2_, *m*_3_}, |**M**| = 3). Let **M**_**o**_ ⊆**M** denote a set of modalities observed by the robot. Active perception is defined as modality selection from a set of unobserved modalities, **M**_***u***_ = **M**\**M**_**o**_, to update the estimation. The 2D space in the upper part of Figure 3 shows the energy distribution in any low-dimensional space (e.g., the principal component (PC) space) of the third hidden layer of the MDBN. Blue indicates lower energy, whereas yellow indicates higher energy. The open circles represent the third hidden activations of the MDBN corresponding to the estimation of the emotion of the partner. The red circle is the ground truth calculated using the signals of all modalities and thus represents the last state of the hidden layer after the robot has received all sensory signals from the interaction partner. Active perception based on energy minimization in the MDBN is performed *T* times (*T* ≦|**M**| − 1) through the following steps.

#### Step 1

The model receives the signal of modality *m*_*init*_ (here, *m*_*init*_ = *m*_2_) as the initial perception and adds the modality to the set of observed modalities ***M***_**o**_. Next, the model estimates the emotion of the partner ***z*** from the observed modality signal [i.e., the white circle $z\left[h_{m_{2}}^{2}\right]$ in the upper part of Figure 3 (Step 1)].

#### Step 2

The second hidden layer reconstructs each unobserved modality feature as a prediction ${\hat{h}}_{m_{n}}^{2}$ (i.e., here ${\hat{h}}_{m_{1}}^{2}$ and ${\hat{h}}_{m_{n}}^{3}$ ) separately from the third hidden layer’s current activation ***z*** $\left[{h^{2}}_{M_{\text{o}}}\right]$ . The model then updates the energy values $E_{m_{n}}$ based on the current observation ${h^{2}}_{M_{\text{o}}}$ , network state ***z*** $\left[{h^{2}}_{M_{\text{o}}}\right]$ , and predicted features of *m*_*n*_ modality ${\hat{h}}_{m_{n}}^{2}$ using Eq. 8.

#### Step 3

The model selects the next perception as the *n*-th modality that minimizes the energy *E*_*n*_ the most from the set of unobserved modalities M_u_ [i.e., *m*_3_ is selected in Figure 3 (Step 3)] and receives the actual signal. The model then adds the modality to set **M**_**o**_ and updates the estimation of the emotion of the partner (i.e., the second white circle).

#### Step 4

The process is repeated from Step 2 until *T* inferences are achieved.

Algorithm 1 provides the details of the procedure. The Monte Carlo sampling number *K* is introduced to calculate the expected energy of each $E_{m_{n}}$ .

Algorithm 1 Active inference based on energy minimization in an MDBN.

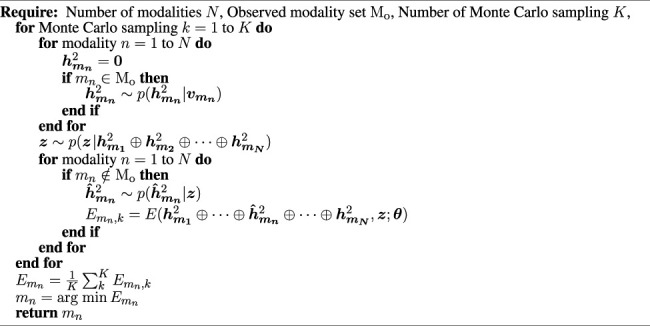

### 4.2 Mathematical Formulation of the Proposed Active Perception Method

To clarify the relationship between the proposed active perception method, the previous method that maximizes information gain (Taniguchi et al., 2018), and active inference (Friston et al., 2017), this section provides a formulation of the proposed method. First, we described the energy of the observed signals and the current estimation as $E^{init}=E\left({h^{2}}_{M_{\text{o}}},z\right)$ and the energy after integrating the predicted modality feature ( ${\hat{h}}_{m_{n}}^{2}$ ) as $E^{pred}=E\left({h^{2}}_{M_{\text{o}}}⊕{\hat{h}}_{m_{n}}^{2},z\right)$ . The proposed method attempts to minimize *E* ^*pred*^. In other words, it attempts to maximize the energy difference between *E* ^*init*^ and *E* ^*pred*^. The energy difference of modality *m*_*n*_ can then be written as follows using Eq. 1.

$$\begin{array}{cc} E_{m_{n}}^{\mathrm{diff}} & =E^{init}-E^{pred}\\ & =\log p\left({h^{2}}_{M_{\text{o}}}⊕{\hat{h}}_{m_{n}}^{2},z\right)-\log p\left({h^{2}}_{M_{\text{o}}},z\right)\\ & =\log\frac{p\left({h^{2}}_{M_{\text{o}}}⊕{\hat{h}}_{m_{n}}^{2},z\right)}{p\left({h^{2}}_{M_{\text{o}}},z\right)}\\ & =\log\frac{p\left(z|{h^{2}}_{M_{\text{o}}},{\hat{h}}_{m_{n}}^{2}\right)p\left({\hat{h}}_{m_{n}}^{2}|{h^{2}}_{M_{\text{o}}}\right)p\left({h^{2}}_{M_{\text{o}}}\right)}{p\left(z|{h^{2}}_{M_{\text{o}}}\right)p\left({h^{2}}_{M_{\text{o}}}\right)}\\ & =\log\frac{p\left(z,{\hat{h}}_{m_{n}}^{2}|{h^{2}}_{M_{\text{o}}}\right)}{p\left(z|{h^{2}}_{M_{\text{o}}}\right)p\left({\hat{h}}_{m_{n}}^{2}|{h^{2}}_{M_{\text{o}}}\right)}+\log p\left({\hat{h}}_{m_{n}}^{2}|{h^{2}}_{M_{\text{o}}}\right) \end{array}$$ (9)

Here, it is supposed that the bias parameter *b*_*j*_ = 0 for all nodes of the second hidden layer of the MDBN.

In Algorithm 1, *K* samples of ***z*** ^[*k*]^ and ${{\hat{h}}_{m}^{2}}^{\left[k\right]}$ are obtained to calculate the expected energy through Monte Carlo sampling. The expected energy difference is expressed as follows.

$$\begin{array}{cc} E\left[E_{m_{n}}^{di\mathrm{ff}}\right] & =\frac{1}{K}\underset{k}{\sum }\log\frac{p\left(z^{\left[k\right]},{{\hat{h}}_{m}^{2}}^{\left[k\right]}|{h^{2}}_{M_{\text{o}}}\right)}{p\left(z^{\left[k\right]}|{h^{2}}_{M_{\text{o}}}\right)p\left({{\hat{h}}_{m}^{2}}^{\left[k\right]}|{h^{2}}_{M_{\text{o}}}\right)}+\frac{1}{K}\underset{k}{\sum }\log p\left({{\hat{h}}_{m}^{2}}^{\left[k\right]}|{h^{2}}_{M_{\text{o}}}\right)\\ & =IG\left(z;{\hat{h}}_{m_{n}}^{2}|{h^{2}}_{M_{\text{o}}}\right)-\left\{-E\left[\log p\left({\hat{h}}_{m_{n}}^{2}|{h^{2}}_{M_{\text{o}}}\right)\right]\right\}. \end{array}$$ (10)

Here, IG denotes the information gain of the prediction ${\hat{h}}_{m_{n}}^{2}$ for the estimation of ***z*** when observation ${h^{2}}_{M_{\text{o}}}$ is given. The first term is similar to the criterion used in the active perception method proposed by Taniguchi et al. (2018). This term also represents the mutual information between the estimation ***z*** and prediction ${\hat{h}}_{m_{n}}^{2}$ . Using this term, our method and the previous technique select the more informative modalities from the unobserved ones based on the prediction.

In contrast, the second term was not included in the previous method. This term represents the negative entropy of the prediction ${\hat{h}}_{m_{n}}^{2}$ conditioned by observations, ${h^{2}}_{M_{\text{o}}}$ . Essentially, it represents the expectation of the likelihood of the prediction ${\hat{h}}_{m_{n}}^{2}$ when the model receives the observation ${h^{2}}_{M_{\text{o}}}$ . If no correlations exist between the multimodal signals, this term is expressed as a constant value for all predictions (i.e., the distribution will be uniform) because the observed signals will have no information for prediction. Meanwhile, if correlations do exist between the multimodal signals, this term will produce different values for predictions, which are made from the same observed signals. We believe that this difference gives our active perception method an advantage over previous methods in emotion recognition. Note that our method does not calculate the information gain and log-likelihood directly. Instead, both values are acquired indirectly by minimizing *E* ^*pred*^.

Next, we compared our method with active inference. The active inference method considers the next action selection to be performed by minimizing the expected free energy $G_{\tau }\left(\pi \right)$ (Friston et al., 2017; Da Costa et al., 2020) in practice. $G_{\tau }\left(\pi \right)$ is expressed as follows.

$$\begin{array}{cc} G_{\tau }\left(\pi \right) & =E_{Q\left(o_{\tau },x_{\tau }|\pi \right)}\left[\ln Q\left(x_{\tau }|\pi \right)-\ln\tilde{p}\left(o_{\tau },x_{\tau }\right)\right]\\ & \approx -\underset{\text{Epistemic Value}}{\underset{︸}{E_{Q\left(o_{\tau }\right)}D_{KL}\left[Q\left(x_{\tau }|o_{\tau }\right)\|Q\left(x_{\tau }|\pi \right)\right]}}+\underset{\text{Extrinsic Value}}{\underset{︸}{\left\{-E_{Q\left(o_{\tau },x_{\tau }|\pi \right)}\left[\ln\tilde{p}\left(o_{\tau }\right)\right]\right\}}}. \end{array}$$ (11)

Here, *o*_*τ*_ and *x*_*τ*_ represent the observations and hidden states at *τ*, respectively, and *π* represents a policy, which is a sequence of actions (i.e., *π* = [*a*_1_, *a*_2_, …, *a*_*τ*_]). Please see the work of Sajid et al. (2020) for a detailed explanation of this equation. The expected free energy here comprises two terms: epistemic and extrinsic values. The epistemic value represents the information gain when the active inference model performs actions using *π* in the future. This term should be maximized to minimize the expected free energy. In other words, the active inference model performs actions to maximize information gain and contributes to reducing the uncertainty in future estimations. The extrinsic value, which includes a minus sign is the log-likelihood of the desired observations *p* (*o*_*τ*_) under the belief in the future. To minimize the expected free energy, the active inference model must minimize this term. This means that the active inference method maximizes the probability of *o*_*τ*_ generated by future actions. According to this characteristic, this term can be described as the model preference. In fact, the first and second terms in Eq. 10 correspond to the terms in Eq. 11. These relations indicate that the proposed method performed energy minimization of the second and third hidden layers of the MDBN (in other words, maximizing the energy reduction in the RBM), which is equivalent to the active inference performed by minimizing the expected free energy.

## 5 Experimental Setup

This section explains the experiments performed to evaluate the performance of the proposed active perception method and its comparison to other methods used in human–robot interaction. We focused on multimodal affective interactions in which attention selection is required. Section 5.1 introduces the multimodal interaction dataset IEMOCAP (Busso et al., 2008) and experimental conditions. Section 5.2 describes the details of the modality signals and the feature extraction method. Finally, Section 5.3 specifies the parameters of the proposed MDBN.

### 5.1 Multimodal Interaction Dataset: IEMOCAP

**FIGURE 4:**
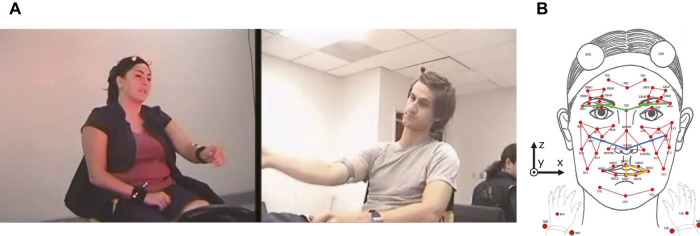
Example interaction data from the IEMOCAP dataset (Busso et al., 2008) and the distances focused as facial movement features.

We employed the IEMOCAP dataset (Busso et al., 2008), which is a multimodal human–human emotional interaction dataset, to train the MDBN and evaluate the proposed active inference method. Figure 4A depicts a sample scene of the IEMOCAP dataset. The dataset comprises approximately 12 h of audiovisual data (motion captures of face and hands and speech) from 10 actors. Their facial expressions and hand movements were recorded using a motion capture system, and the conversations were recorded using additional video cameras. Fifty-three and six motion capture markers were attached to the faces and hands of the actors, respectively (Figure 4B), while communicating with other actors. During the interaction process, the actors expressed many types of emotional states based on the scenario and circumstances of the interaction process.

All recorded data were segmented into utterances, and three evaluators were used to annotate each utterance using an emotion label. The set of emotional labels contained nine states: happiness, excitement, surprise, neutral, frustration, anger, sadness, fear, and disgust. We selected the category with the majority vote as the ground truth of the emotional state for each utterance. If two or more categories had the same number of votes, we set the data category to “ambiguous state.” As a result, each utterance was assigned one of the 10 emotional labels. Our final dataset contained 4,985 utterances; Table 1 lists the number and percentage of each emotional utterance.

**TABLE 1:** Amount and percentage of emotional data.

Emotional labels	Number of data	Percentage [%]
Happiness	297	5.96
Excitement	549	11.00
Surprise	31	0.62
Neutral	606	12.20
Frustration	998	20.00
Anger	621	12.40
Sadness	653	13.10
Fear	20	0.40
Disgust	1	0.02
Ambiguous	1,209	24.30
Total	4,985	100.00

To evaluate the performance of our method in situations with different levels of difficulty, we designed three cases, including a 10% case, in which 90% of the data were used for training and 10% for testing, a 30% case, in which 70% of the data were used for training and 30% for testing, and finally, a novel person case, in which the data from nine randomly selected actors were used for training and the remaining data were used for testing. The test dataset in the third condition was very unfamiliar to the model compared to those in the first two conditions. In each situation, we produced 10 dataset variations to enable statistical analysis.

### 5.2 Feature Extraction From Audiovisual Signals

We obtained multimodal emotion expressions from each utterance. The audiovisual data were divided into seven modalities (i.e., |**M**| = 7). The first five modalities contained the visual information regarding the movements of the right hand (*m*_1_), left hand (*m*_2_), mouth (*m*_3_), cheek (*m*_4_), and eyebrow (*m*_5_). The two audio modalities included the pitch and intensity of the vocalization (*m*_6_) and its mel-frequency cepstral coefficient (MFCC) (*m*_7_). We extracted statistical features from the modalities mentioned above as input signals of the MDBN, as follows.

First, we obtained modality-dependent features. The hand movement features (i.e., *m*_1_ and *m*_2_) consisted of the velocity and acceleration of two markers in each hand, where the velocity and acceleration were measured in three dimensions (i.e., the x, y, and z dimensions). As a result, the gesture modalities had 12 dimensions each. The facial movement features (i.e., *m*_3_, *m*_4_, and *m*_5_) consisted of the distances between markers in each region and their derivatives. We focused only on several motion capture markers, as shown in Figure 4B. The mouth movement was measured as three distances indicated using the yellow lines in Figure 4B. The cheek movement (*m*_4_) was represented by the two distances indicated in blue, and the eyebrow movement (*m*_5_) consisted of the four distances colored in green. Each distance was normalized using the distance between the eyes of the individual (i.e., the intra-person distance) and represented in a two-dimensional (x-z) space because the y-coordinates of the markers did not change significantly. Finally, *m*_3_, *m*_4_, and *m*_5_ had 12, 8, and 16 dimensions, respectively. The first audio features (*m*_6_) consisted of pitch, intensity, and their time differences from the prior time step. The second audio features (*m*_7_) consisted of 13-dimensional MFCCs and their time differences. The audio modalities had 4 and 26 dimensions, respectively.

Next, we calculated the statistical values of each feature during each utterance. The statistical values included the mean, variance, range, maximum, and minimum values of each feature. We defined these statistical values as the input signals of each modality-specific DBN. Ultimately, the numbers of dimensions for *m*_1_, *m*_2_, *m*_3_, *m*_4_, *m*_5_, *m*_6_, and *m*_7_ were 60, 60, 60, 40, 80, 20, and 130, respectively.

### 5.3 Network Structure and Training Method

The proposed MDBN consisted of seven modality-specific DBNs and one additional hidden layer. Each modality-specific DBN had three layers (visible, first hidden, and second hidden layers). The number of visible units of each modality-specific DBN was set to the number of dimensions corresponding to the input signals. Specifically, the RBMs of *m*_1_, *m*_2_, *m*_3_, *m*_4_, *m*_5_, *m*_6_, and *m*_7_ had 60, 60, 60, 40, 80, 20, and 130 visible units, respectively, and the number of the first hidden units in each network was set to half the number of visible units up to a maximum of 50, i.e., 30, 30, 30, 20, 40, 10, and 50, respectively. The number of the second hidden layer units was set to 10 in each case to avoid the imbalance of information between modalities. The third hidden layer was connected to the second hidden layers of all the modality-specific DBNs, as shown in Figure 2B. This RBM received inputs that were concatenated outputs from all the modality-specific DBNs. Therefore, there were 70 units in the second hidden layer. Finally, the third hidden layer had 20 hidden units. All the connecting weights of the networks were initialized using normal distributions with a mean value of zero and a unit variance. We constructed the MDBN using our full scratched program^1^.

The MDBN was trained using the training dataset corresponding to each situation (i.e., 10%, 30%, and novel person cases). First, each modality-specific DBN was trained separately. Next, the third hidden layer was trained by concatenating the output (i.e., the second hidden layer) of the modality-specific DBNs. Every RBM was trained for 10,000 steps in an unsupervised manner (i.e., the MDBN did not use the emotional labels for training).

## 6 Experimental Results

The purpose of these experiments was to verify how the proposed active perception method performed in the proposed MDBN. Therefore, we first evaluated the detailed behavior and process of the proposed active perception method by selecting one modality signal from the test datasets as initial modality (i.e., **M**_**o**_ = *m*_*init*_ in the first step of active inference). The MDBN was trained using the training datasets corresponding to each scenario as explained in Section 5.1. Section 6.1 describes the acquired multimodal representation in MDBN and the state transitions through active perception under the 10% case.

Next, we employed additional neural networks to estimate emotional states from the multimodal representations and compared the change in the estimation accuracy through active perception with the existing methods under all of the dataset cases in Section 6.2. Finally, Section 6.3 discusses the implications of the results. We set the number of Monte Carlo samples as *K* = 100, and the active perception evaluations were performed 10 times under each condition for statistical analysis in all experiments.

It is to be noted that all active perception methods were evaluated in the test phase of the emotion estimation task (i.e., after the model was trained).

### 6.1 Result I: Active Perception Using the Proposed Method

**FIGURE 5:**
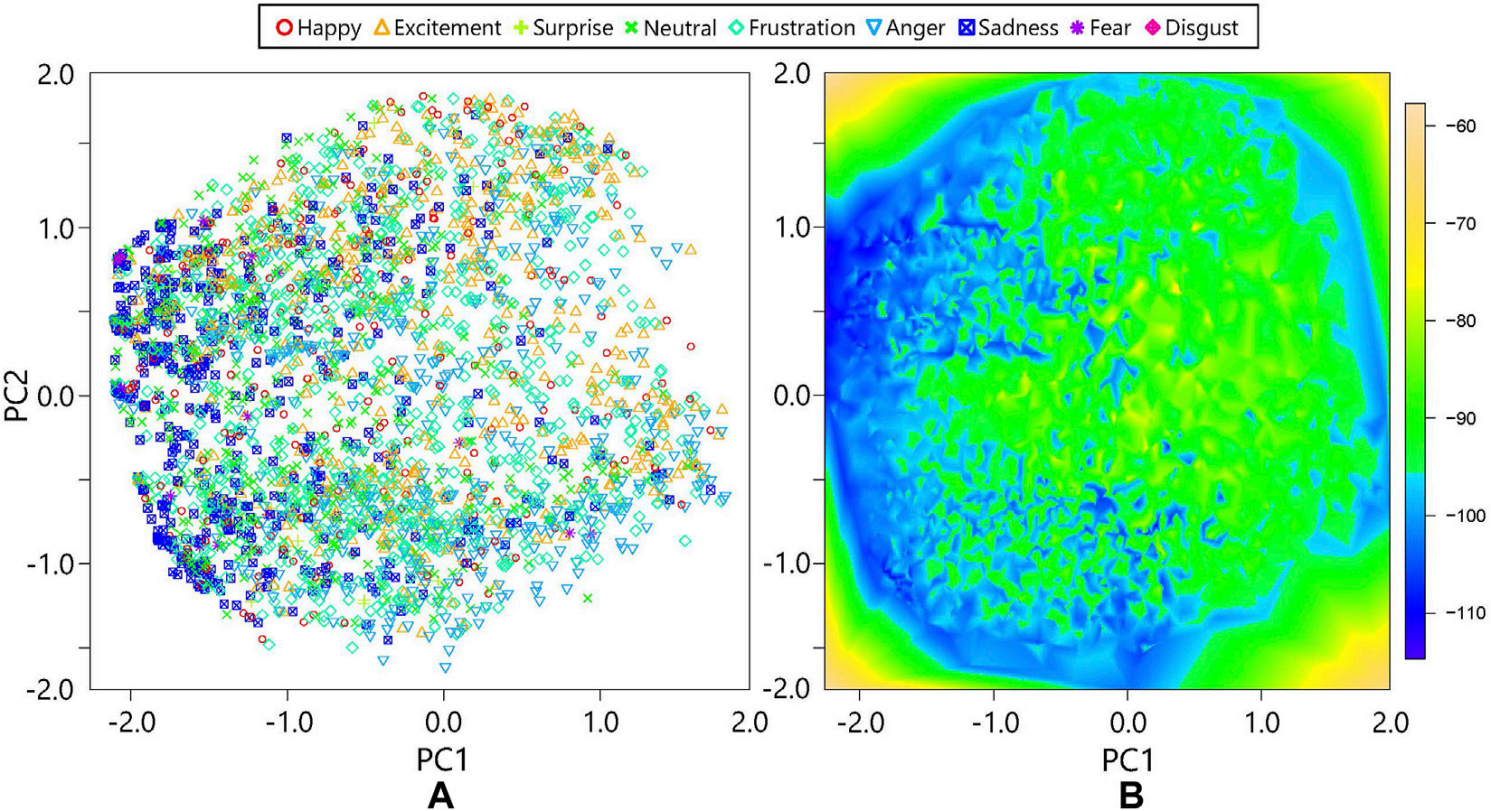
Representations and energies in the first and second PC spaces: **(A)** activations of the third hidden layer of the MDBN with emotional labels; **(B)** energy distribution of hidden activations.

First, we verified the distribution of multimodal emotional expressions and their energy values in the MDBN. We performed PC analysis for the 20-dimensional outputs of the third hidden layer to visualize the representation in a 2D space, as shown in Figure 3. Figure 5 depicts the first and second PC spaces of the third hidden layer’s activations of the MDBN. Each marker in the left graph indicates the activation when the model used the signals of all the modalities. The colors and shapes of the markers represent the emotional categories of the multimodal data, where those of ambiguous states were omitted. Note that the MDBN did not use the emotional labels in training. Although many emotional categories are widely distributed in the PC space, the neutral, sad, and fear categories have bias. Specifically, their expressions were placed on the left side of the PC space. The *x*-axis (i.e., PC 1) represents the intensity of the multimodal expressions because the emotional categories mentioned above have lower intensities. In contrast, PC 2 represents the individual characteristics, where the emotional categories are uniformly distributed on this axis. The colors in the right graph represent the distributions of the energy values in the same PC space. A lower energy values corresponds to a higher probability of the data in the energy-based models. The left side of the graph shows low energies because the data are concentrated in this region. The energy distribution in the PC space is not smooth because the PC transformed the data representation linearly. However, the distribution in the original space (i.e., 20-dimensional hidden activation) may be smooth.

**FIGURE 6:**
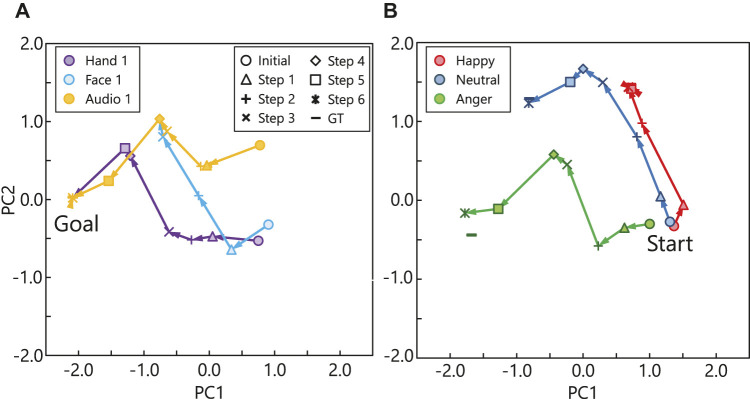
Example transitions of hidden activations in PC space through active inference: **(A)** transitions of estimation using different initial modalities; **(B)** transitions of estimation for different emotions.

Next, we provide two examples of emotion estimation through active perception. Figure 6A shows the transition of emotion estimation in the PC space. The same emotional state was estimated using different modalities as the initial modality (i.e., Hand 1: *m*_1_, Face 1: *m*_3_, and Audio 1: *m*_6_). Each color and marker represents the initially observed modality and number of active perceptions, respectively. The ground truth calculated using all modality signals is represented by ∗. The emotional state of this particular expression is “sad”. Each estimation started from a different initial modality and traversed to the ground truth stepwise through active perception. The transitions of the Face 1 (*m*_3_) and Audio 1 (*m*_6_) conditions overlap starting from Step 5 because the same modalities were selected in Steps 5 (*m*_2_, left hand) and 6 (*m*_1_, right hand). This occurred because the hands did not always move actively because the actors were sitting on chairs. Therefore, the hand movement conveyed less information than the other modalities, and thus, it was selected later in the active perception. The modalities selected through active perception are listed in Table 2. Figure 6B shows the results of active perception for different emotional expressions. In each case, Audio 1 (*m*_6_) was the initial modality, where the audio signals are similar to each other. The emotional states corresponding to these three cases include happy, neutral, and angry, which are represented by different colors. The transition in the estimation for the happy expression reaches the ground truth faster than it does in the other cases. Two interesting findings can be derived from these results. First, only a few modalities represent the happy state: Audio 1 (*m*_6_), Audio 2 (*m*_7_), Face 1 (*m*_3_), and Face 2 (*m*_4_). Therefore, our active perception method could efficiently select highly informative modalities. Second, the anger and neutral emotions require more steps to be recognized accurately because anger is usually difficult to distinguish from the other negative emotions, such as, frustration and disgust. In addition, the estimation of the neutral expression had an error compared to the ground truth because the probability of hidden activation had a wider distribution (i.e., high entropy) owing to the inherent characteristics of the neutral emotion.

**TABLE 2:** Order of modalities selected through active perception under each condition.

	Different initial modality			Different emotion		
	**Hand 1**	**Face 1**	**Audio 1**	**Happy**	**Neutral**	**Anger**
*m*_*init*_	*m*_1_	*m*_3_	*m*_6_	*m*_6_	*m*_6_	*m*_6_
1st	*m*_7_	*m*_7_	*m*_7_	*m*_7_	*m*_7_	*m*_7_
2nd	*m*_6_	*m*_6_	*m*_5_	*m*_3_	*m*_5_	*m*_3_
3rd	*m*_5_	*m*_4_	*m*_4_	*m*_4_	*m*_4_	*m*_4_
4th	*m*_4_	*m*_5_	*m*_3_	*m*_5_	*m*_3_	*m*_5_
5th	*m*_3_	*m*_2_	*m*_2_	*m*_1_	*m*_2_	*m*_2_
6th	*m*_2_	*m*_1_	*m*_1_	*m*_2_	*m*_1_	*m*_1_

### 6.2 Result II: Quantitative Evaluation of the Proposed Method Compared to Other Methods

We evaluated how the accuracy of emotion estimation increased through active perception. We employed a four-layered feed-forward neural network (FFNN) to estimate emotional categories from the multimodal representations of human expressions that are activations of the third hidden layer of the MDBN. The number of nodes per layer was 20 (i.e., the number of the third hidden layer’s units of the MDBN), 64, 32, and 8 (i.e., the number of emotional categories, excluding disgust and ambiguous states) from the input layer to the softmax layer (i.e., the top layer). All layers, except the softmax layer, used a rectified linear unit (ReLU) function as the activation function. We used the Keras library (Chollet, 2015) to build this network and the RMSprop as an optimizer. The FFNN learned the relationships between the MDBN outputs and their emotional categories in a supervised manner. However, the connection weights of the MDBN were not fine-tuned through the FFNN training process. In other words, each network was trained independently. We compared the estimation accuracy of our method to that of two other methods: the IG.max and random methods. The IG.max approach is an active perception method based on the information gain maximization proposed by Taniguchi et al. (2018). We used information gain maximization instead of energy minimization as the active perception criterion of the MDBN. We set the number of Monte Carlo samples as *K* = 100. The random strategy involved selecting a modality *m*_*n*_ from ***M***_***u***_ randomly at each step. All methods were evaluated in the three dataset cases: the 10%, 30%, and novel person cases described in Section 5.1.

**FIGURE 7:**
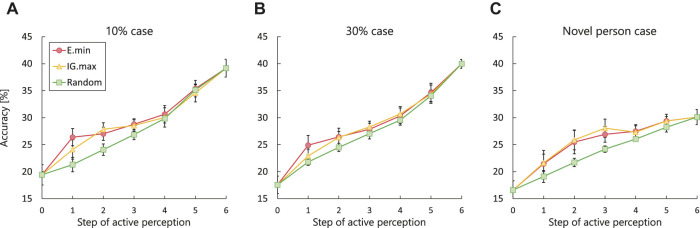
Change in estimation accuracy using each active perception method in all dataset cases: **(A)** estimation accuracy in the 10% case; **(B)** estimation accuracy in the 30% case; **(C)** estimation accuracy in the novel person case.

Figure 7A–C and Table 3 show the changes in the estimation accuracy^2^ for each of the three cases and the results of statistical analysis. The different colors indicate the results for the different methods. The accuracy in the initial and final steps is the same for all the active perception methods under each set of conditions. The maximum estimation accuracy was approximately 40% when the model used all the modality signals (see Section 6.3.2 for further discussion). The experimental results show that the estimation accuracy of the random method increases linearly through active perception. In contrast, E.min and IG.max exhibit significant increases in estimation accuracy at an early stage of active perception.

**TABLE 3:** Change in estimation accuracy mean and standard deviation through each active perception method in all dataset cases. The values in parentheses indicate the standard deviation.

	10% case			30% case			Novel person case		
	E.min	IG.max	Random	E.min	IG.max	Random	E.min	IG.max	Random
initial	19.43 (1.88)	19.43 (1.88)	19.43 (1.88)	17.55 (1.63)	17.55 (1.63)	17.55 (1.63)	16.59 (1.71)	16.59 (1.71)	16.59 (1.71)
1st	**26.39(1.59** )***^,†††^	**24.11(1.79)** ^†††^	21.31 (1.34)	**24.90(1.82**)**^,†††^	**22.90(1.27)** ^†^	21.79 (0.62)	**21.46**(**2.47**)^†^	**21.62**(**1.62**)^†††^	19.10 (1.11)
2nd	**27.02(1.23)** ^†††^	**27.85(1.21)** ^†††^	24.08 (1.06)	**26.44(1.62)** ^†††^	**26.36(1.11)** ^†††^	24.50 (0.76)	**25.48**(**2.18**)^†††^	**25.87**(**1.83**)^†††^	21.71 (0.77)
3rd	**28.77**(**1.03**)^†††^	**28.54(1.07)** ^†††^	26.87 (0.93)	27.85 (1.13)	**28.28(1.07)** ^†^	27.04 (0.98)	**26.91**(**1.48**)^†††^	**28.05**(**1.70**)^†††^	24.17 (0.59)
4th	30.65 (1.63)	30.06 (1.79)	29.85 (0.84)	30.33 (1.45)	**30.68(1.37)** ^†^	29.54 (0.93)	**27.48**(**1.23**)^†††^	**27.31**(**1.19**)^†††^	26.04 (0.42)
5th	35.38 (1.52)	34.59 (1.65)	35.15 (0.93)	34.64 (1.73)	34.35 (1.70)	34.05 (1.06)	**29.38**(**0.81**)^††^	**29.37**(**1.25**)^†^	28.23 (0.89)
6th	39.17 (1.65)	39.17 (1.65)	39.17 (1.65)	39.97 (0.82)	39.97 (0.82)	39.97 (0.82)	30.10 (1.42)	30.10 (1.42)	30.10 (1.42)

*(*p* < 0.05), **(*p* < 0.01), ***(*p* < 0.005): significant difference from IG.max.

^†^(*p* < 0.05), ^††^(*p* < 0.01), ^†††^(*p* < 0.005): significant difference from Random.

**FIGURE 8:**
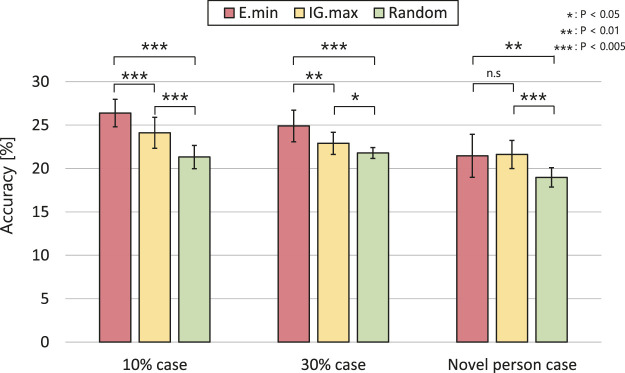
Estimation accuracy for the first active perception executed in each dataset case.

Figure 8 highlights the estimation accuracy in the first step of active perception. We conducted a Student’s t-test for each set of conditions. In the novel person situation, the results of the proposed and IG.max methods show no significant difference: *t* (18) = 1.734, *p* = 0.430. In contrast, their results exhibit significant differences in the 10 and 30% cases: *t* (18) = 1.734, *p* < 0.005 and *t* (18) = 1.734, *p* < 0.01, respectively, although both methods outperformed the random approach.

These experimental results demonstrate that the active perception methods using information criteria can update their estimations more accurately by obtaining more informative signals when the robot has limited resources for paying attention to human expressions. In particular, our method outperformed the IG.max approach in the first step of active perception, and the performance difference was more significant in the 10% case than in the 30% case. Meanwhile, there were no significant differences between the proposed and IG.max methods from the second step of active perception (see Table 3). We conclude that our method achieved improved accuracy faster than the IG.max method using limited information in this task.

### 6.3 Discussion

#### 6.3.1 Critical Differences Between the Energy Minimization and the Information Gain Maximization

In previous studies on object category estimation (Sakaguchi, 1993; Taniguchi et al., 2018), mutual information between the current estimation and unobserved modality signals has been used as a criterion for active perception. Such methods chose the next modality whose expectation of mutual information is the highest amongst the unobserved modalities. This strategy corresponds to information gain maximization because mutual information represents the amount of information between two random variables. Therefore, the previous method and the IG.max approach considered in our experiment can be regarded as techniques that only consider the first term in Eq. 10 for active perception. In contrast, our method selects the next modality indirectly based on energy minimization, considering both terms in Eq. 10. The difference between the proposed and previous methods is the second term in Eq. 10. This term, which is the expectation of the negative likelihood of the prediction, represents the negative entropy of the predicted modality signal conditioned by the current observation. Moreover, it takes a higher value when the probability of the prediction becomes uniform. Specifically, this term can be minimized if the system has knowledge of the prediction and/or the multimodal signals are related (i.e., correlated).

This advantage of the proposed method is demonstrated in the experimental results presented in Figure 8. In the novel person case, the MDBN could not model the probability of unobserved modality signals, $p\left({\hat{h}}_{m_{n}}^{2}|{h^{2}}_{M_{\text{o}}}\right)$ , because the test data consisted of unknown actors. Therefore, the second term in Eq. 10 provided little to no information for modality selection (i.e., uniform distribution). As a result, the proposed method and the IG.max approach show similar results in this case. In contrast, the 10 and 30% cases revealed the advantage of the proposed method. The MDBN could properly estimate the probability of the test data because the model captured the tendencies of the emotional expressions of all the actors by detecting the correlation between multimodal expressions. The difference between the two methods is larger in the 10% case than in the 30% case. This result indicates that the second term in Eq. 10 models relationships between multimodal emotion expressions accurately using numerous training data and provides a considerable amount of information for modality selection. In other words, the proposed method has an advantage over the IG.max method when the knowledge of the MDBN overlaps with the test situation.

#### 6.3.2 Current Limitations and Future Challenges

In these experiments, we assumed that the proposed active perception and other methods could obtain information from the partner without any cost during the interaction. In addition, we assumed that the emotional expressions of the partner did not change until all multimodal signals were acquired. However, in a real HRI context, the robot would expend resources to obtain observations, and the partner’s emotional state dynamically changes over time during the interaction. It is necessary to consider the number of constraints to conduct active perception (i.e., the maximum number of active perceptions) during the interaction. Improving the proposed method to take the robot’s resources, such as the cost of behavior that acquires the information and constraints on the number of active perceptions for determining the estimation of other’s emotions (i.e., decision-making) into account will be explored in the future studies.

Recent studies that recognize emotional states from the IEMOCAP dataset achieved approximately 70% accuracy (Tripathi et al., 2018, 2019). In comparison with these studies, our results show no advantages in the emotion recognition task because the maximum accuracy of emotion estimation in our experiments was about 40% when the robot used complete observations (i.e., all modality signals). We believe that the results may be attributed to two issues; the training process of the MDBN and the emotion estimation model. The training process of the MDBN and the FFNN were separated to shield the structure of the MDBN energy function (i.e., model parameters) from the supervised training of the FFNN. Therefore, the MDBN could not obtain an effective representation for the emotion estimation in FFNN. Additionally, the network structure of the estimation module (i.e., the FFNN) was more straightforward than that of other networks used in previous studies (e.g., convolutional neural networks (CNN), recurrent neural networks (RNN), and long short-term memory (LSTM)). However, we focused on verifying the characteristics of the proposed method rather than improving the accuracy of emotion estimation in this study. To achieve higher estimation accuracy for practical use in actual HRI, we intend to explore not only to use time series models such as LSTM but also to apply the proposed active perception method to a model that integrates the representation learning and recognition into a single energy-based model in future research.

## 7 Conclusion

In this study, we have proposed an active perception method based on energy minimization in an MDBN. The key concept underlying the proposed method involves obtaining the next sensation by selecting the modality for minimizing the network energy. The energy of the model represents the likelihood of the corresponding network state. Therefore, our method involves selecting the most plausible modality based on the current estimation. First, we formulated the proposed method and compared it with other active perception methods, i.e., methods considering information gain (Taniguchi et al., 2018) and the active inference technique proposed by Friston et al. (2017) based on the free energy principle. Next, we applied the active perception methods in an emotion estimation task assuming affective communication between a human and a robot. The methods were compared to each other in three dataset cases with different balances between the training and test datasets. When the training dataset contained more of the same characteristics as the test dataset, our active perception method achieved significantly improved accuracy than the other methods in the test phase using limited information. This result indicates that the additional term in our formulation (i.e., the second term in Eq. 10), which is the likelihood of predictions, provides an advantage when the network can capture the relationships between multimodal signals, and the robot can select informative modality expressions from the human to estimate their emotions with limited resources. We conclude that our method, which is analogous to active inference, incorporates and even extends the previous methods that assumed modality independence. In our future research, we intend to evaluate the performance of our method in practical situations. For example, the emotion of the partner changes during interaction, and the robot needs to pay a price to obtain perceptions. In addition, we intend to apply the proposed method to actual robot tasks for affective communication.

## Data Availability

Publicly available datasets were analyzed in this study. This data can be found here: https://sail.usc.edu/iemocap/.

## Footnotes

1
https://github.com/takato1414/rbm_sets.git

2 We also calculated the macro-F1 score because of the imbalance of emotional classes in the dataset. The trend of the results was the same in both cases; therefore, we show only micro-F1 scores (i.e., accuracy) for easy comparison of our work with other studies.
